# Time-modified OSEM algorithm for more robust assessment of left ventricular dyssynchrony with phase analysis in ECG-gated myocardial perfusion SPECT

**DOI:** 10.1186/s40658-019-0261-z

**Published:** 2019-12-27

**Authors:** Matti J. Kortelainen, Tuomas M. Koivumäki, Marko J. Vauhkonen, Mikko A. Hakulinen

**Affiliations:** 1 Department of Applied Physics, University of Eastern Finland, POB 1627, FI-70211 Kuopio, Finland; 2 Diagnostic Imaging Center, Kuopio University Hospital, Kuopio, Finland; 3 Department of Medical Physics, Central Finland Central Hospital, Jyväskylä, Finland

**Keywords:** SPECT, Myocardial perfusion imaging, Phase analysis, Dyssynchrony, Image reconstruction

## Abstract

**Background:**

In ordered subsets expectation maximization (OSEM) reconstruction of electrocardiography (ECG)-gated myocardial perfusion single-photon emission computed tomography (SPECT), it is often assumed that the image acquisition time is constant for each projection angle and ECG bin. Due to heart rate variability (HRV), this assumption may lead to errors in quantification of left ventricular mechanical dyssynchrony with phase analysis. We hypothesize that a time-modified OSEM (TOSEM) algorithm provides more robust results.

**Methods:**

List-mode data of 44 patients were acquired with a dual-detector SPECT/CT system and binned to eight ECG bins. First, activity ratio (AR)—the ratio of total activity in the last OSEM-reconstructed ECG bin and first five ECG bins—was computed, as well as standard deviation SD_R-R_ of the accepted R–R intervals; their association was evaluated with Pearson correlation analysis. Subsequently, patients whose AR was higher than 90% were selected, and their list-mode data were rebinned by omitting a part of the acquired counts to yield AR values of 90%, 80%, 70%, 60% and 50%. These data sets were reconstructed with OSEM and TOSEM algorithms, and phase analysis was performed. Reliability of both algorithms was assessed by computing concordance correlation coefficients (CCCs) between the 90% data and data corresponding to lower AR values. Finally, phase analysis results assessed from OSEM- and TOSEM-reconstructed images were compared.

**Results:**

A strong negative correlation (*r* = -0.749) was found between SD_R-R_ and AR. As AR decreased, phase analysis parameters obtained from OSEM images decreased significantly. On the contrary, reduction of AR had no significant effect on phase analysis parameters obtained from TOSEM images (CCC > 0.88). The magnitude of difference between OSEM and TOSEM results increased as AR decreased.

**Conclusions:**

TOSEM algorithm minimizes the HRV-related error and can be used to provide more robust phase analysis results.

## Background

Maximum likelihood expectation maximization (MLEM) algorithm and its accelerated variant—ordered subsets expectation maximization (OSEM) algorithm—are currently the recommended reconstruction methods in clinical myocardial perfusion single-photon emission computed tomography (SPECT) imaging [1]. These algorithms permit taking into account several physical factors that affect image acquisition—most notably photon attenuation and scattering, and collimator-detector response (CDR). Yet another factor is image acquisition time per projection angle [2]; however, this factor may be ignored in the reconstruction process because usually fixed acquisition time per projection angle is used.

However, in electrocardiography (ECG)-gated myocardial perfusion SPECT, a common method to perform ECG gating is fixed forward gating [3, 4], in which each R–R interval is divided into bins of fixed length (usually 8 or 16 bins) starting from the leading R wave. As the duration of the R–R interval often varies during image acquisition due to heart rate variability (HRV), the R–R intervals shorter than the average duration provide less emission data to the image corresponding to the last ECG bin. Effectively, the last ECG bin is acquired with a shorter total acquisition time. This “data shortage” may also vary between the projection angles. If not accounted for in the reconstruction, the image corresponding to the last ECG bin inevitably appears as having lower activity than the images corresponding to the first ECG bins.

Phase analysis is a relatively novel tool to quantify left ventricular (LV) mechanical dyssynchrony. It is based on dividing LV myocardium into a number of smaller regions and approximating their time–activity curves (TACs) by fitting first Fourier harmonic functions to the time–activity data sampled at each region through the cardiac cycle [5]. Subsequently, phase angles of these fitted TACs are calculated and binned into a histogram whose width is used as a measure of LV mechanical dyssynchrony. Therefore, if activity in the image corresponding to the last ECG bin is significantly lower than in the other ECG bins, this may cause errors in the fit and consequently skew the final phase analysis results [6].

In this paper, we have three hypotheses. First, we hypothesize that patients with a larger degree of HRV are associated with a larger degree of data shortage in the last ECG bin. Second, we hypothesize that incorporation of projection angle and ECG bin-specific acquisition time into the OSEM algorithm enhances the robustness of phase analysis; we call this modification a time-modified OSEM algorithm (TOSEM). Third, we hypothesize that phase analysis results assessed from TOSEM-reconstructed images differ from those obtained from OSEM-reconstructed images, and that the magnitude of this difference is related to the degree of data shortage in the last ECG bin.

## Materials and methods

### Study population and image acquisition

The study population consisted of 44 patients (19 female) referred to standard stress/rest 1-day myocardial perfusion SPECT study. Their (mean ± standard deviation) characteristics were as follows: age, 68 ± 10 years; height, 169 ± 10 cm; weight, 78 ± 13 kg; body mass index, 27.4 ± 4.1 kg/m^2^. Each patient received 300 MBq of Tc-99m tetrofosmin before the stress imaging, followed by 705 ± 12 MBq before the rest imaging 3 h later according to our institutional guidelines. To ensure clinical workflow, data were collected during the rest phase only for this study. Written informed consent was obtained from all patients and the study was approved by the Research Ethics Committee of the Northern Savo Hospital District (Dno 90/2011; March 20, 2012).

The patients were imaged in a supine position with a dual-detector SPECT/CT system (Precedence; Koninklijke Philips N.V., Amsterdam, Netherlands). The following SPECT list-mode image acquisition protocol was used: 90° detector configuration, low-energy high-resolution collimators, noncircular detector orbit, 64 projection angles from the right anterior oblique to the left posterior oblique, acquisition time of 30 s per projection angle, energy window of 140 keV ± 10%, and ECG gating acceptance window of ±20%. R triggers for ECG gating were generated using Cardiac Trigger Monitor 3000 unit (Ivy Biomedical Systems, Inc., Branford, CT, USA). For off-line list-mode data processing, we also recorded a signal-form ECG with a data acquisition system (MP150 and ECG100C; BIOPAC Systems, Inc., Goleta, CA, USA).

The list-mode data were binned into ECG-gated projection images using custom-made MATLAB scripts (MATLAB R2015b; The MathWorks, Inc., MA, USA). Matrix size of 96×96 and pixel size of 6.22 mm were used. ECG gating was realized as fixed forward gating, dividing R–R intervals into eight ECG bins; the length of each bin was calculated as one-eighth of the average duration of those R–R intervals that were accepted in the ECG gating.

### Reconstructions

Reconstructions were carried out in MATLAB environment. Rotation-based reconstruction approach was adopted and the rotation matrices were computed using Gaussian interpolation [7]. In forward and backward projections, the CDR was modeled as a distance-dependent Gaussian function [8]. For both OSEM and TOSEM reconstructions, ten iterations and eight subsets were used, as suggested in previous studies [9, 10]. Reconstructed transaxial images were rotated into short-axis images, smoothed with a three-dimensional Gaussian filter with a standard deviation of 1 voxel [11] and masked to eliminate extracardiac activity.

The general OSEM/TOSEM iterative algorithm is presented in matrix format in Algorithm 1. In Algorithm 1, ***f̂***^(n)^ denotes the image vector at update number *n*, ***H*** denotes the transition matrix, ***g*** denotes the measured projection image vector, ***1*** denotes a vector of ones, *T* denotes matrix transpose, *S*_*n*_ denotes the *n*th ordered subset, *N* denotes the number of subsets and *M* denotes the number of iterations. The notation “*ℑ*∈*S*_*n*_” indicates that only the matrix/vector rows that belong to subset *S*_*n*_ are used in the calculation. See [12].

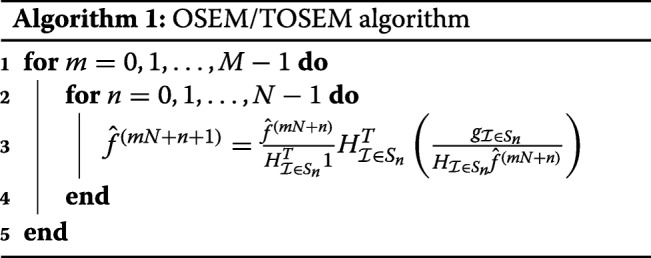



In our case, the transition matrix is formed of matrix blocks ***H***^*l*^, *l* = 1,…,*L*, where *L* is the number of projection angles. That is,

$$ \boldsymbol{H}={\left[{\left[{\boldsymbol{H}}^1\right]}^T,\dots, {\left[{\boldsymbol{H}}^L\right]}^T\right]}^T. $$

In the standard OSEM algorithm, an individual matrix block is

$$ {\boldsymbol{H}}^l={\boldsymbol{P}}^l{\boldsymbol{R}}^l, $$

where ***P***^*l*^ and ***R***^*l*^ are the forward projection matrix (including CDR modeling) and rotation matrix at projection angle *l*, respectively. In the TOSEM algorithm, the only difference compared to OSEM is that we add to each transition matrix block a term *τ*^*l*^, which is the duration of projection angle *l* [2], to obtain

$$ {\boldsymbol{H}}^l={\tau}^l{\boldsymbol{P}}^l{\boldsymbol{R}}^l. $$

**Fig. 1:**
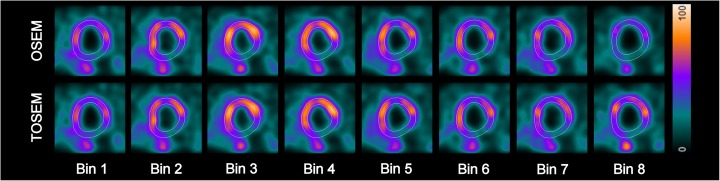
Short axis views of ECG-gated patient SPECT data reconstructed with OSEM and TOSEM algorithms as displayed by Quantitative Gated SPECT 2012 program. It can be seen that the eighth bin in the OSEM-reconstructed image series has a lower activity level compared to other bins due to heart rate variability. This phenomenon is not present in the TOSEM-reconstructed image series

Reconstructing each ECG bin individually with TOSEM results in a series of images where the total activity in the image remains constant from one ECG bin to another. This is not necessarily the case with the standard OSEM reconstruction due to the lack of duration factors *τ*^*l*^ in the transition matrix, as shown in Figure 1.

### Phase analysis

Phase analysis was performed with Quantitative Gated SPECT (QGS) 2012 program (Cedars-Sinai Medical Center, Los Angeles, CA, USA). In phase analysis, QGS samples the LV myocardial activity from up to 1008 sampling points [13] from all ECG bins to form time–activity curves (TACs) for each sampling point. These TACs are further interpolated by fitting first Fourier harmonic (FFH) function to the time–activity data. The amplitude and phase angle of each FFH are recorded, and 5% of the data that correspond to the lowest amplitudes are discarded [14]. The remaining phase angles are used to build a 360-bin histogram, which is characterized by computing the histogram bandwidth (BW), phase angle standard deviation (StD) and entropy (ENT) [14].

### Specific studies

In the first part of the study, we assessed the association between the HRV and the data shortage in the last ECG bin. To do this, we computed the standard deviation SD_R-R_ of those R–R intervals that were accepted in the ECG gating. This parameter was further normalized by dividing with the average of the accepted R–R intervals. The parameter describing the data shortage in the last ECG bin, the activity ratio (AR), was computed by summing the voxel values of OSEM-reconstructed images at each ECG bin to provide a value for total activity and calculating the ratio of the total activity in the last ECG bin and the average total activity in the first five ECG bins [6]. The association between SD_R-R_ and AR was evaluated by computing their Pearson correlation coefficient (*r*).

In the second part of the study, we assessed the robustness of phase analysis results when AR was artificially reduced. Having computed the AR for all 44 patients, we selected those 14 patients whose AR was larger than 90% [6]. For these patients, we rebinned the list-mode data such that a part of the last ECG bin data was discarded uniformly from all projection angles to yield AR values of 90%, 80%, 70%, 60% and 50%. These data were reconstructed with both OSEM and TOSEM algorithms. Phase analysis was performed on all reconstructed images.

Statistical analysis was performed in SPSS Statistics v.23 (IBM Corporation, NY, USA). Sphericity of phase analysis data was assessed with Mauchly’s test. Repeated measures analysis of variance (ANOVA) with post hoc pairwise multiple comparison tests (Bonferroni corrected) was performed to assess whether there were significant (*p* < 0.05) differences between images of different AR values. Reliability was assessed by computing Lin’s concordance correlation coefficient (CCC) between the 90% data and the lower AR values. The CCC values were computed in MATLAB environment as [15]

$$ CCC=r\bullet {C}_b=\frac{\sigma_{xy}}{\sigma_x{\sigma}_y}\bullet \frac{2{\sigma}_x{\sigma}_y}{\sigma_x^2+{\sigma}_y^2+{\left({\mu}_x-{\mu}_y\right)}^2}, $$

where *r* is the Pearson’s correlation coefficient, *C*_*b*_ is the bias correction factor, *σ*_*xy*_ is the covariance of data vectors *x* and *y*, *σ*_*x*_ and *σ*_*y*_ are the standard deviations of data vectors *x* and *y*, respectively, and *μ*_*x*_ and *μ*_*y*_ are the means of data vectors *x* and *y*, respectively.

In the third and final part of the study, we assessed how much phase analysis results change with respect to AR in the whole study population when the images are reconstructed with TOSEM instead of OSEM. Data from all 44 patients were reconstructed with both OSEM and TOSEM algorithms and subjected to phase analysis. Pearson correlation coefficients were computed between AR and the changes of phase analysis parameters (∆BW, ∆StD and ∆ENT).

## Results

### First part of the study

**Fig. 2:**
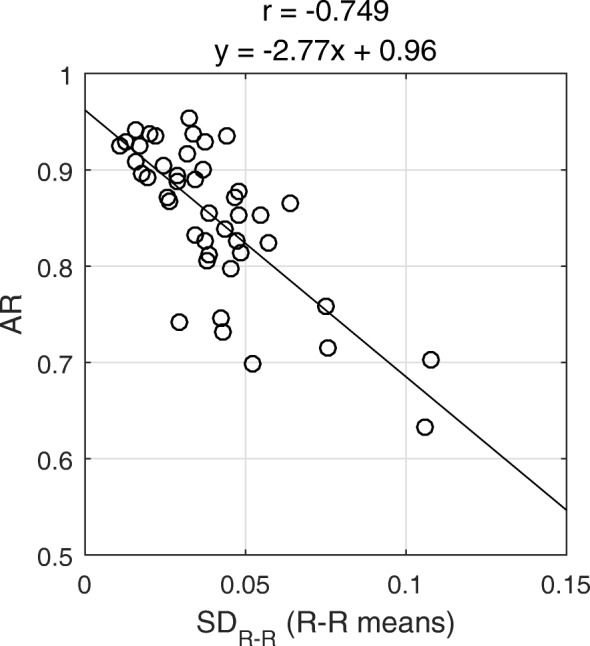
Scatter plot of the R–R interval standard deviation (SD_R-R_) vs. activity ratio (AR). Pearson’s correlation coefficient (r) and regression line equation are displayed

A strong negative correlation (*r* = -0.749, *p* < 0.001) was found between SD_R-R_ and AR: the larger the SD_R-R_, the smaller was the AR (Figure 2). This means that patients with larger R–R length variation are associated with larger degrees of data shortage in the last ECG bin.

### Second part of the study

According to Mauchly’s test, BW, StD and ENT data violated the assumption of sphericity regardless of whether OSEM or TOSEM algorithm was used (*p* < 0.05). Therefore, we report the Greenhouse–Geisser corrected ANOVA test results (with associated sphericity values (*ε*)).

For OSEM algorithm, there were significant differences between different AR values for BW (*ε* = 0.275, *F*(1.101, 14.310) = 28.313, *p* < 0.001), StD (*ε* = 0.265, *F*(1.060, 13.778) = 21.098, *p* < 0.001) and ENT (*ε* = 0.380, *F*(1.521, 19.778) = 123.500, *p* < 0.001). All pairwise comparisons between different AR values were statistically significant (*p* < 0.05) for all phase analysis parameters. The average values of all phase analysis parameters decreased as the AR value decreased (Table 1). According to the CCC values, reliability of all phase analysis parameters decreased considerably as the AR value decreased (Table 2).

**Table 1:** Phase analysis results for patients whose unaltered activity ratio was > 90%

	OSEM			TOSEM		
AR (%)	BW (°)	StD (°)	ENT (%)	BW (°)	StD (°)	ENT (%)
90	41.6 ± 15.2	14.1 ± 6.3	57.4 ± 5.3	49.9 ± 20.9	17.4 ± 7.6	59.6 ± 5.2
80	35.5 ± 10.9	11.5 ± 4.8	56.2 ± 4.9	49.3 ± 19.3	17.5 ± 7.3	59.8 ± 5.2
70	32.2 ± 9.4	9.6 ± 3.6	54.9 ± 4.9	49.6 ± 19.4	17.2 ± 7.0	59.6 ± 5.1
60	29.0 ± 8.0	8.0 ± 2.7	53.4 ± 4.8	51.2 ± 20.1	17.5 ± 7.2	59.6 ± 5.3
50	27.0 ± 7.1	7.2 ± 2.1	52.5 ± 4.7	48.5 ± 17.1	16.9 ± 6.9	59.9 ± 4.9

The results are expressed as mean ± standard deviation

AR activity ratio, BW bandwidth, StD standard deviation, ENT entropy

**Table 2:** Concordance correlation coefficients for OSEM reconstructions for patients whose unaltered activity ratio was > 90%

AR (%)	BW			StD			ENT		
90 vs.	CCC	*C*_*b*_	*r*	CCC	*C*_*b*_	*r*	CCC	*C*_*b*_	*r*
80	0.820	0.853	0.961	0.856	0.870	0.984	0.970	0.974	0.996
70	0.653	0.700	0.933	0.578	0.630	0.918	0.879	0.889	0.989
60	0.473	0.536	0.882	0.279	0.407	0.685	0.729	0.756	0.965
50	0.362	0.433	0.836	0.168	0.289	0.582	0.653	0.671	0.973

AR activity ratio, BW bandwidth, StD standard deviation, ENT entropy, CCC concordance correlation coefficient, *C*_*b*_ bias correction factor, *r* Pearson’s correlation coefficient

For TOSEM algorithm, there were non-significant differences between different AR values for BW (*ε* = 0.395, *F*(1.581, 20.558) = 0.926, *p* = 0.391), StD (*ε* = 0.391, *F*(1.564, 20.327) = 0.463, *p* = 0.589) and ENT (*ε* = 0.521, *F*(2.083, 27.083) = 0.533, *p* = 0.600) (Table 1). According to the CCC values, reliability was very good (CCC > 0.88) for all phase analysis parameters regardless of which AR value was compared to the 90% data (Table 3).

**Table 3:** Concordance correlation coefficients for TOSEM reconstructions for patients whose unaltered activity ratio was > 90%

AR (%)	BW			StD			ENT		
90 vs.	CCC	*C*_*b*_	*r*	CCC	*C*_*b*_	*r*	CCC	*C*_*b*_	*r*
80	0.988	0.997	0.992	0.978	0.999	0.978	0.993	0.999	0.994
70	0.938	0.997	0.941	0.962	0.996	0.966	0.993	1.000	0.993
60	0.966	0.997	0.969	0.976	0.998	0.978	0.979	1.000	0.979
50	0.889	0.977	0.910	0.899	0.993	0.906	0.970	0.997	0.973

AR activity ratio, BW bandwidth, StD standard deviation, ENT entropy, CCC concordance correlation coefficient, *C*_*b*_ bias correction factor, *r* Pearson’s correlation coefficient

#### Third part of the study

**Fig. 3:**
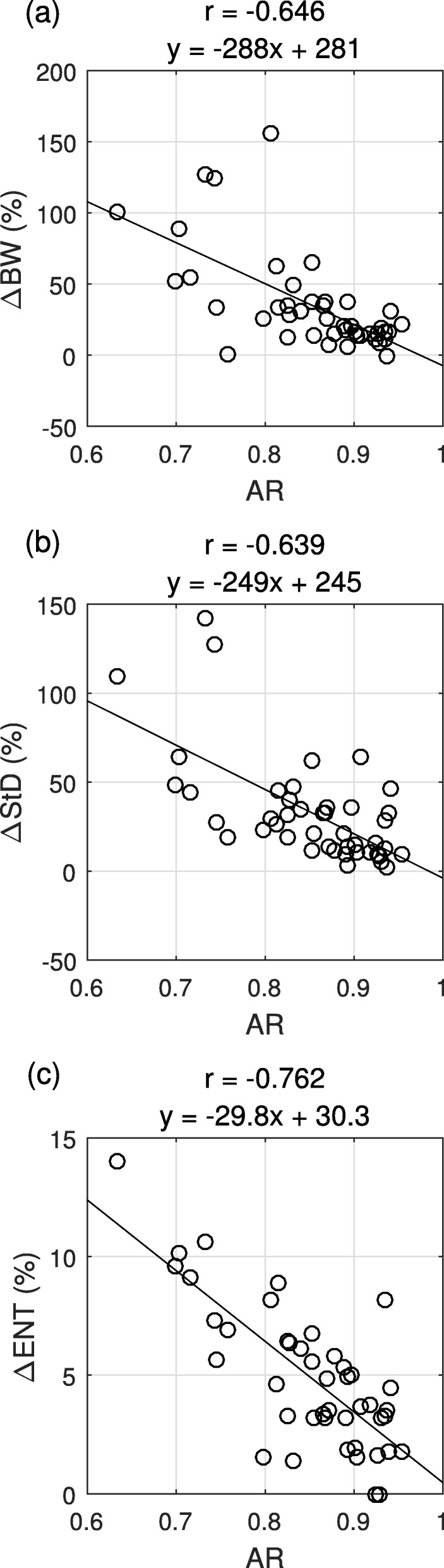
Scatterplots of activity ratio (AR) vs. changes of bandwidth (∆BW), standard deviation (∆StD) and entropy (∆ENT) between OSEM and TOSEM. Pearson’s correlation coefficients (*r*) and regression line equations are displayed

**Fig. 4:**
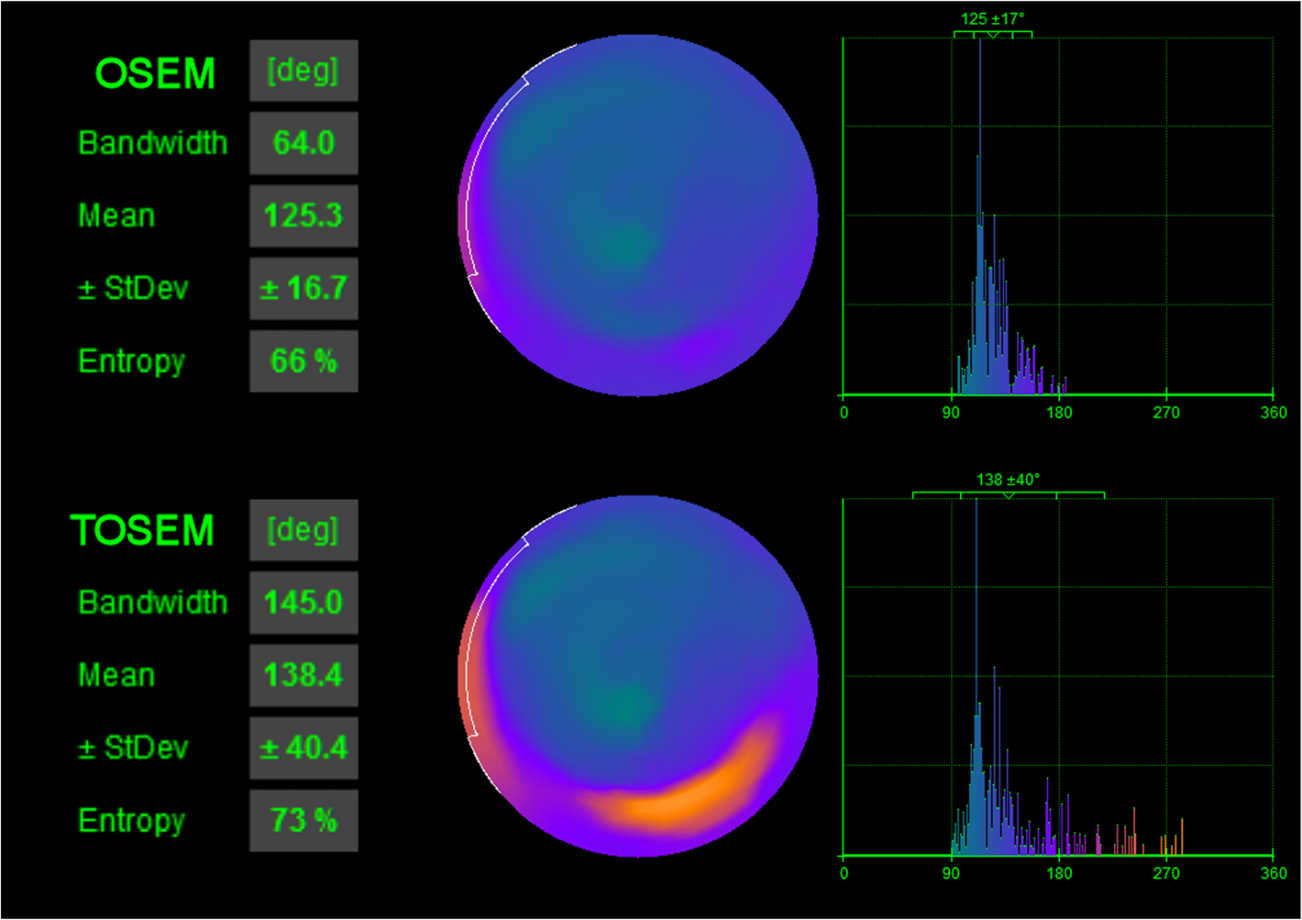
Phase analysis results from Quantitative Gated SPECT 2012 program for the same patient as in Figure 1. Phase analysis assessed from OSEM-reconstructed images yielded mildly abnormal results, with the polar map showing relatively well-synchronized function. On the contrary, phase analysis assessed from TOSEM-reconstructed images yielded considerably abnormal results, with the polar map clearly showing delayed contraction in the inferior basal region of the left ventricle. For this patient, the calculated activity ratio value was 73%

The differences between phase analysis parameters assessed from OSEM and TOSEM were statistically significant according to paired-samples *t* test (*p* < 0.001) with TOSEM always providing greater or equal parameter values. Moderate negative correlations were found between AR and ∆BW (*r* = -0.646, *p* < 0.001) and between AR and ∆StD (*r* = -0.639, *p* < 0.001). In addition, a strong negative correlation was found between AR and ∆ENT (*r* = -0.762, *p* < 0.001). Thus, the larger the AR, the smaller was the difference between the parameters assessed from OSEM- and TOSEM-reconstructed images (Fig. 3). This indicates that the greater degrees of data shortage (smaller AR value) are associated with a greater difference between OSEM- and TOSEM-assessed phase analysis parameters, with TOSEM providing greater parameter values. An example of phase analysis results is shown in Fig. 4.

## Discussion

Incorporation of projection angle-specific acquisition time factors into MLEM/OSEM reconstruction is not a novel idea; it was noted approximately 35 years ago [2] and has since been applied in, e.g., respiratory motion-corrected reconstruction [16]. However, as far as we know, this modification and its effects have not been considered before in the context of LV mechanical dyssynchrony assessment with phase analysis.

Our results indicated that there was a strong negative association between the R–R interval length variation, SD_R-R_ and the computed AR value; however, we expected to find even stronger association. This discrepancy is possibly related to the characteristics of OSEM reconstruction. The AR value is not affected by the average number of measured counts in all projection images of the last ECG bin, but rather by the average number of measured counts in *the last ordered subset* of the last ECG bin. As a very simplified example, if the last ordered subset consisted only of blank measured projection images, the image estimate would be multiplied with zero after the OSEM backprojection step, and the final reconstructed image would also be blank, regardless of how many counts were measured in the projection images of the other subsets. That is, the AR value is effectively computed based on data from only a fraction of all projection angles, whereas SD_R-R_ is computed based on data from all projection angles. Therefore, it is reasonable that the correlation coefficient between SD_R-R_ and AR was smaller than we anticipated. Nevertheless, these results confirmed our first hypothesis that patients with a larger degree of HRV suffer from a larger degree of data shortage in the last ECG bin.

Subsequently, we simulated lower AR values in 14 patients by rebinning the list-mode data and omitting certain proportion of the measured counts. The resulting data were reconstructed with both OSEM and TOSEM algorithms and phase analysis was performed on the reconstructed images. The results were clear: when AR value decreased, the phase analysis parameters assessed from OSEM-reconstructed images also decreased, whereas the parameters assessed from TOSEM-reconstructed images retained the original values assessed with AR value of 90% (Table 1). According to the calculated CCC values, the reliability of TOSEM results was superior to that of OSEM results (Tables 2 and 3). These results confirmed our second hypothesis that TOSEM is more robust than OSEM to the phenomenon of data shortage of the last ECG bin associated with HRV.

Finally, we investigated whether there is, in general, a difference in phase analysis results between OSEM- and TOSEM-reconstructed images and whether this difference depends on the calculated AR value. The associations between AR and the changes of the phase analysis parameters were all at least moderate in strength (*r* < -0.50), indicating the presence of significant relationships. These results confirmed our third hypothesis that TOSEM-assessed phase analysis results differ from OSEM-assessed results and that the magnitude of this difference is related to the degree of data shortage in the last ECG bin.

Surprisingly, the changes in phase analysis results (∆BW, ∆StD and ∆ENT) were relatively large (Figure 3). An AR value of 80%, which, according to this study population, is likely to occur in clinical studies, was associated with over 5% increase of ENT, over 45% increase of StD and over 50% increase of BW when the data were reconstructed with TOSEM instead of OSEM. In the study of Hämäläinen et al., QGS phase analysis normal reference values were derived from a study population of 52 patients without cardiac diseases [17]; the 95th percentile values for BW, StD and ENT were 63.7°, 26.5° and 63.7%, respectively, at rest. Comparing our results to these reference values, 7 patients out of 44 turned from normal to abnormal based on BW, 6 patients based on StD and 10 patients based on ENT, when the more robust reconstruction method (TOSEM) was used. Obviously, Hämäläinen et al. reconstructed their data using the OSEM algorithm [17], which denotes that the data shortage effect may have skewed their results; the reference values could have actually been higher if they had reconstructed their data using the TOSEM algorithm.

This study had some limitations. First, the study population was relatively small and heterogeneous; however, it represented an unselected, real patient population. Second, we were unable to make head-to-head comparison between OSEM and TOSEM at AR value of 100%. These two reconstruction methods should, in theory, lead to similar phase analysis results when there is no data shortage phenomenon involved. However, the highest unaltered AR value we observed was 95%, and this was for only one patient, so verifying this point was impossible. Third, phase analysis could be performed on only one commercial software, the QGS. Different myocardial perfusion image analysis softwares differ, for example, by the way they locate the myocardial surfaces and sample the myocardial activity [18]. In addition, there may be differences in how the phase analysis itself is carried out—for example, multiple harmonics could be used instead of just the first Fourier harmonic [19].

The disadvantage of TOSEM algorithm in the context of ECG-gated reconstruction is, of course, that it requires knowledge of both projection angle and ECG bin-specific acquisition times. This would practically necessitate access to list-mode data, which could render the algorithm inaccessible for some laboratories. However, several SPECT camera manufacturers (Philips, Siemens, GE, Mediso, and Spectrum Dynamics Medical) support data recording in list-mode format, so we do not expect this to become an issue. Alternatively, one could request the camera manufacturer to alter the SPECT camera’s binner software to output an array of acquisition times in addition to projection images, thus enabling the use of the TOSEM algorithm.

Instead of the TOSEM algorithm, one could also use the OSEM algorithm and retrospectively scale the total image activity in the last ECG bins to the level of the first ECG bins. This is the approach applied by Ludwig et al. [6]. However, one might want to be prudent in scaling the activity as the authors themselves left the last ECG bins unaltered if their relative activity was higher than 90% [6]. In case of TOSEM, no retrospective scaling is necessary as the shorter total acquisition time of the last ECG bins is accounted for in the reconstruction and thus they are automatically scaled to correct activity levels.

HRV generally decreases with age and development of cardiac diseases [20]; therefore, one could infer that typical patients undergoing myocardial perfusion SPECT (old and suspected to have cardiac diseases) do not suffer from HRV-induced artifacts. Our results indicate this is not the case: even for our patient with the lowest SD_R-R_ (0.011, or 9.1 ms) the AR value was only 92.5%, which is sufficiently low to induce artifacts to phase analysis assessed from OSEM-reconstructed images (Figure 3). As AR value decreases further due to a larger degree of HRV, the changes in phase analysis parameters become significantly greater. Therefore, as some degree of HRV is inevitable during myocardial perfusion SPECT imaging, we recommend reconstructing the patient data with TOSEM algorithm if phase analysis is required.

## Conclusions

The degree of data shortage in the last ECG bin is strongly associated with heart rate variability. Phase analysis assessed from TOSEM-reconstructed images is more robust to the data shortage effect than phase analysis assessed from OSEM-reconstructed images. If phase analysis is required, we recommend reconstructing the data with the TOSEM algorithm.

## Data Availability

Data will not be shared because it will be used in the ongoing PhD project.
